# Morphology, Taxonomy, Geographic Distribution, Genetic Diversity, and Phylogenomics of the Genus *Tulipa* L.: A Comprehensive Review

**DOI:** 10.3390/plants15121817

**Published:** 2026-06-12

**Authors:** Nazerke Aiture, Ashimkhan Kanayev, Roza Mussina, Damet Kyzdarova, Gulzhanat Sultangaliyeva, Zagipa Sapakhova

**Affiliations:** 1 Faculty of Biology and Biotechnology, Al-Farabi Kazakh National University, Almaty 050040, Kazakhstan; nazerkeaiture1998@gmail.com (N.A.); asimhankanaev4@gmail.com (A.K.); gulzhanat.sm98@gmail.com (G.S.); 2 Faculty of Biology and Geography, Buketov Karagandy National Research University, Karagandy 100024, Kazakhstan; rozamusina8@gmail.com (R.M.); dametkenkizdarova@gmail.com (D.K.); 3 Breeding and Biotechnology Laboratory, Institute of Plant Biology and Biotechnology, Almaty 050040, Kazakhstan

**Keywords:** biodiversity of *Tulipa*, geographic diversity, taxonomic databases, population structure, phylogenetic analysis, plastome-based genomics, conservation

## Abstract

The genus *Tulipa* L. is a common group of ornamental plants, characterized by high morphological variability and a complex taxonomy. Despite considerable interest in this group, assessments of its species composition remain inconclusive, as evidenced by discrepancies between contemporary taxonomic sources. The number of recognized taxa varies across major taxonomic databases, including Plants of the World Online, World Flora Online, and Euro+Med PlantBase, reflecting ongoing taxonomic revisions and differences in species concepts. In terms of distribution patterns, 7.6% are widely distributed taxa across transcontinental regions, 28.0% occur across multiple countries within a continent, and 66.9% are range-restricted taxa. The latter group includes 4.2% transnational endemics, 44.1% single-country endemics, 8.5% single-region endemics, and 10.2% single-site endemics. Recent taxonomic and evolutionary studies of *Tulipa* increasingly rely on molecular approaches, particularly DNA barcoding and chloroplast genome analyses, which have improved phylogenetic resolution and species delimitation in several cases. However, truly comprehensive studies combining morphological, cytogenetic, and molecular datasets remain limited and are typically restricted to individual taxa or species complexes rather than the genus as a whole. Modern molecular genetic studies demonstrate the high informativeness of both nuclear and plastid markers for studying the phylogeny, systematics, and genetic diversity of *Tulipa* species. Natural populations of *Tulipa* are under pressure from anthropogenic factors and climate change, resulting in reduced range and habitat degradation. According to the International Union for Conservation of Nature Red List of Threatened Species, among 118 taxa of the genus *Tulipa*, *T. sprengeri* Baker is classified as Extinct in the Wild, 5.9% as Critically Endangered, 5.9% as Endangered, 8.5% as Vulnerable, 11.9% as Near Threatened, and 11.0% as Least Concern. The use of exclusively national assessments to determine species extinction risk may be insufficiently objective, whereas global assessments provide a more informative and reliable approach for evaluating conservation status. In this review, we combine investigations of the morphology, taxonomy, and geographic diversity; population genetic structure and molecular diversity; and molecular phylogenetics and plastome-based genomics of the genus *Tulipa*. Furthermore, the review examines current challenges and future research prospects, emphasizing that studies of the genus *Tulipa* should integrate morphological, genomic, and ecological approaches to refine taxonomy and conserve genetic resources.

## 1. Introduction

The genus *Tulipa* L. (family *Liliaceae*) is one of the most popular and economically significant ornamental bulbous plants worldwide, with high commercial value [1]. *Tulipa* species are widely used in horticulture and ornamental landscaping in Asia, Europe, and North Africa [2]. *Tulipa* species are cultivated worldwide as ornamental plants, and their wild relatives represent important genetic resources for breeders, particularly in the context of climate change [3].

The greatest species diversity is concentrated in Central Asia [4,5,6,7,8], followed by South-West Asia and the Middle East, the Caucasus and Eastern Europe, the Mediterranean region, and East Asia. Most species are restricted to mountain ecosystems and occur at altitudes of 700–2200 m, ranging from widely distributed to narrowly endemic taxa [9,10,11,12], including from widely distributed species to several different countries to range-restricted species such as transnational endemics shared between national borderlines of neighboring countries, single-country endemics, or single-region endemics within national territories.

Historically, *Tulipa* became widely known in Europe in the 16th century following its introduction from the Mediterranean region and the Ottoman Empire. The French diplomat and naturalist Pierre Belon (1517–1564) may have been among the first to introduce *Tulipa* to Europe. In 1553, he described red lilies growing in Ottoman gardens [13]. According to historical sources, the first *Tulipa* introduced into Europe belonged to the *Tulipa gesneriana* L. group, which was widely cultivated in Ottoman gardens. Bailey [14] reported that Gesner first observed a *Tulipa* in Augsburg in 1559, while Bailey [14] and Hall [15] suggested that modern cultivated *Tulipa* originated from the *T. gesneriana* group. Linnaeus subsequently classified all garden *Tulipa* spp. under this name. Some authors have also proposed the involvement of *T. suaveolens* Roth, originally recorded in southern Europe, although its native status in the region remains uncertain [14,16]. One of the best-known ornamental species to spread across Europe was *T. sylvestris* L.—the so-called wild *Tulipa* [17]. The first bulbs were introduced from Bologna (northern Italy) and Montpellier (southern France) during the 1550s–1570s. Several prominent botanists, including Gessner, Wieland, Aldrovandi, de Lobel, Clusius, and Dodoens, contributed to the introduction of these plants. The well-developed Flemish botanical network of the sixteenth century played an important role in the introduction and naturalization of *T. sylvestris* throughout Europe [17].

According to various studies, the genus *Tulipa* may comprise around 150 taxa [2,11,18]. At the same time, some authors suggest that there are around 76 species, ranging from south-western Europe and North Africa to Central Asia [12,19]. The taxonomy and classification of the genus *Tulipa* have long been the subject of scientific debate [19]. As reported by Kubentayev et al. [12], the total number of *Tulipa* species may range from 90 to 120 taxa, depending on the taxonomic approach used. Furthermore, several new species of the genus *Tulipa* have been described in recent years [20].

The number of recognized species also varies depending on the taxonomic databases used [21,22,23]. The results of a comparative analysis of taxonomic databases demonstrate varying degrees of consistency in their species composition. At the same time, the overlap among the three databases is limited, reflecting differences in coverage comprehensiveness and taxon inclusion criteria [21,22,23]. According to the *Tulipa* classification and the IUCN Red List of Threatened Species assessments, a significant proportion of species fall into high-risk categories, including rare and threatened taxa [24]. This emphasizes the importance of their conservation and monitoring. Overall, the taxa of the genus are characterized by pronounced chorological heterogeneity: both widespread species and forms with restricted ranges are present. The latter predominate and include transnational endemics, species restricted to specific countries or regions, and narrowly localized and locally distributed taxa. These discrepancies indicate the persistence of taxonomic inconsistencies among international botanical databases and highlight the need for further systematic and phylogenetic studies of the genus *Tulipa*.

According to the taxonomic databases, the highest number of species is found in Kazakhstan and Uzbekistan, with 31 species each [21,25]. A slightly smaller number of species has been recorded in Kyrgyzstan (28), Iran (24), and Tajikistan (21). Eighteen species have been recorded in Turkey, 13 in China, 12 in Afghanistan [21], and 11 in the Balkan regions (Greece) [23].

The genus *Tulipa* is characterized by high species diversity and a wide geographical distribution [4,7,19,21]. The main center of *Tulipa* diversity is in Central Asian countries, including Kazakhstan, Uzbekistan, Kyrgyzstan, and Tajikistan [4,12,26,27]. Furthermore, many *Tulipa* species are threatened with extinction, underscoring the need for further study, inventory, and conservation [3,28]. The cultivation of some species in Europe, North America, and other countries poses a risk of genetic introgression with wild populations [17,29,30]. Furthermore, detailed mapping of their distribution remains limited, and studies on the ecoregional and phytogeographic distribution of species are underrepresented [11]. Comprehensive studies of *Tulipa* species are necessary because the genus is taxonomically complex and cannot be reliably delineated solely by morphological features [31,32,33]. Although enough molecular markers are currently available [25,34,35,36,37,38,39,40], their application to resolving species-level relationships and the molecular taxonomy of *Tulipa* species remains limited and often uncertain. Comparative studies of *Tulipa* plastid genomes remain underrepresented in the scientific literature [1,2,18,41,42,43,44,45], limiting understanding of their evolutionary relationships and genetic diversity. In this regard, research on the species diversity, distribution, taxonomy, and status of natural *Tulipa* populations is vital for conserving genetic resources. Moreover, expanded research on *Tulipa* species is necessary to assess genetic variability, clarify phylogenetic relationships, identify new informative molecular markers, and develop effective conservation strategies for rare and endangered *Tulipa* species.

The aim of this review is to summarise current data on the taxonomic complexity of the genus *Tulipa*, analyze the use of morphological and molecular approaches in studying its species, and assess the role of plastid genomes and molecular markers in clarifying phylogenetic relationships and improving the molecular taxonomy of the genus. The potential applications of these data for studying genetic diversity and developing conservation strategies for rare and endangered *Tulipa* species are also discussed.

## 2. Morphology of the Genus *Tulipa* L.

The development of perennial bulbous geophytes characterizes the genus *Tulipa*, which has an annually regenerating bulb composed of a basal plate and fleshy scales covered by a tunic [31]. The bulb serves as an organ for storing nutrients and vegetative reproduction, forming daughter bulbs, and in some species, stolons, which facilitate the spatial dispersal of populations [15,46].

*Tulipa* leaves are widely strap-shaped and cauline. The above-ground part comprises one to several linear-lanceolate leaves, arranged at the base or along the stem [31,47]. Leaf shapes can be oval, elliptical, or equilateral, but are always lanceolate. Plants typically have two to six leaves, most commonly two to four (e.g., *T. iliensis* Regel, *T. alberti* Regel, *T. aleppensis* Boiss. ex Regel, *T. altaica* Pall. ex Spreng., *T. anisophylla* Vved., *T. annae* J.de Groot & Zonn., *T. armena* Boiss., *T. cretica* Boiss. & Heldr., *T. foliosa* Stapf tulip examples), or five to seven (e.g., *T. tetraphylla* Regel, *T. akamasica* Christodoulou, Hand & Charalamb.), rarely solitary (e.g., *T. regelii* Krasn.). Arrangement ranges from closely clustered to scattered along the stem. Leaf blades vary from linear (e.g., *T. heteropetala* Ledeb., *T. sylvestris*, *T. linifolia* Regel, *T. serbica* Tatic & Krivošej), and linear-lanceolate (e.g., *T. albanica* Kit Tan & Shuka, *T. uniflora* Besser ex Baker, *T. scardica* Bornm., *T. undulatifolia* Boiss.) or narrowly lanceolate (e.g., *T. kaufmanniana* Regel, *Tulipa sprengeri* Baker) to broadly lanceolate or sublorate (e.g., *T. tetraphylla*, *T. alberti*) [7,21,48,49,50]. Leaf orientation varies among taxa, with leaves being erect-ascending (sect. *Lanatae*), spreading (e.g., *T. borszczowii* Regel, *T. iliensis*), or occasionally prostrate on the soil surface (e.g., *T. annae*, *T. altaica*). In some species, leaves are densely aggregated, in others loosely or widely spaced. Leaf surface varies from glabrous (e.g., *T. albanica*, *T. uniflora*) to pubescent (e.g., *T. borszczowii*, *T. altaica*, *T. heteropetala*), smooth to slightly crisp; often glaucous, giving a bluish-green appearance. In some taxa, the adaxial surface is pubescent. Leaf colour ranges from predominantly green to reddish-green (e.g., *T. annae*), sometimes brownish or whitish- to reddish-margined (e.g., *T. uniflora*, *T. greigii* Regel). In certain species, the upper surface bears anthocyanin spotting (sect. *Vinistriatae* (Van Raamsd.) Zonn.) or lacks such markings (sect. *Lanatae*). Leaf margins are generally entire, occasionally slightly undulate or curled. In some taxa, leaves are weakly keeled or have longitudinal ridges. Leaf length is variable, with leaves either reach the level of the flower (e.g., *T. borszczowii*) or exceed it (e.g., *T. lehmanniana* Merckl) [4,12].

The stem is erect and robust, ranging from 7 to 75 cm in length, depending on the species and growing conditions [47]. It is smooth and cylindrical, with a bright green coloration. The stem is unbranched and somewhat succulent, with a thick, fleshy texture that helps to store water. Leaves clasp the stem, creating a sturdy structure, and there are no thorns or spines. Overall, the stem is well-adapted for supporting the plant’s upright growth [12].

*Tulipa* flowers are typically solitary and erect, ranging from campanulate (bell-shaped) to cup-shaped forms, varying greatly in color and shape like cup, bowl, or star, depending on the cultivar or species [47]. The flower is pentacyclic, that is, it consists of five whorls and consequently comprises two pairs of three tepals each: inner and outer; six anthers in two whorls; and a single carpel. They range from plain white and pink to yellow, orange, and red; there are many shades between these colors—blue and black do not exist [46,47]. Some *Tulipa* species are multi-flowered, mainly those of the subgenus *Eriostemones* Boiss., as well as some of the subgenus *Orithyia* (D. Don) Baker, whilst those of the subgenera *Tulipa* and *Clusianae* (Baker) Zonn. are mainly single-flowered [31]. The length of the stamens may vary, and their number equals the number of tepals. The colour of the spots, the margin, the shape, and the basal coloration of the flowers may vary not only between species or populations, but also from the outer to the inner tepals, and even from one side of a petal to the other [31]. Flowers usually solitary or 1–2 (rarely up to 4, e.g., *T. tetraphylla*, *T. turkestanica* (Regel) Regel, *T. praestans* H.B.May), actinomorphic, campanulate to star-shaped, relatively large to medium-sized. Perianth composed of 6 free tepals, highly variable in colour, ranging from white, yellow, and red to bicoloured or multicoloured forms, with or without a distinct basal blotch. In several species, flowers show diagnostic characters: nodding in bud and at anthesis (e.g., *T. bifloriformis* Vved., syn. *T. orthopoda* Vved.); whitish with a yellow blotch (e.g., *T. biflora* Pall., syn. *T. buhseana* Boiss.); pale yellow or whitish (e.g., *T. dasystemon* (Regel) Regel); tepals with a dark violet blotch on both surfaces (e.g., *T. borszczowii*); basal blotch mainly on inner tepal surface (e.g., *T. lehmanniana*); tepals shortly pubescent at base, dull pinkish-red with yellow margins (e.g., *T. annae*); outer tepals yellowish-grey with pinkish suffusion (e.g., *T. altaica*). Flowers yellow with tepals often incurved (e.g., *T. lemmersii* Zonn., Peterse & J.de Groot), or yellow with straight tepals and orange-yellow filaments (e.g., *T. kolpakowskiana* Regel). Tepals red (e.g., *T. ostrowskiana* Regel), yellow-red, or yellow, usually with a black or yellow basal blotch (e.g., *T. korolkowii* Regel; *T. lehmanniana*, syn. *T. zenaidae* Vved.; *T. greigii*). Inner tepals obovate and slightly longer than outer tepals (e.g., *T. greigii*), or triangular-obovate and shorter than outer tepals (e.g., *T. alberti*). Tepals obtuse to subobtuse with filaments gradually attenuate from the base (e.g., *T. uniflora*), whereas tepals very acute with filaments broadened below the middle and anthers up to 9 mm long (e.g., *T. heteropetala*). Flowers yellow, often with violet tinging on outer tepals (e.g., *T. sylvestris* subsp. *australis* (Link) Pamp., syn. *T. biebersteiniana* Klokov & Zoz), and white with yellow base and greenish-grey outer tepals (e.g., *T. patens* Schult. f.) [4,12].

The fruit of *Tulipa* is a leathery capsule, varying from ellipsoid to subglobose in shape and distinctly 3-angled. At maturity, the capsule dehisces loculicidally, splitting longitudinally along the middle of each valve. Numerous flattened seeds are arranged in two rows within each locule of the capsule. Seeds serve as the principal means of sexual reproduction in the genus. Fruit colour usually changes during maturation, becoming yellowish- to brownish-green and finally brown at seed dispersal stage [47]. In addition, *Tulipa* species are defined based on capsule characteristics, including length, diameter, and shape [31].

A study of seeds from eight species in China has revealed differences in seed morphology and size. In *T. edulis* (Miq.) Baker, the seeds are crescent-shaped with an invisible embryo and longitudinal striations, whereas in the other species, the seeds are sector-shaped with a visible linear embryo and a reticulate surface pattern; seed size also varied [32]. A study of the seed morphology of *T. patens*, *T. altaica*, *T. biflora*, *T. uniflora*, and *T. heteropetala* from Eastern Kazakhstan showed that they differ qualitatively not only in size and weight, but also morphologically in terms of such characteristics as the micropyle, chalazal end, seed scar, rafe, endosperm, and embryo. Differences in seed material were clearly evident in two closely related species from the *Orithyia* section: *T. uniflora* and *T. heteropetala* [51].

*Tulipa* spp. are capable of sexual reproduction through pollination, followed by seed formation [4]. Apomixis has also been documented in *Tulipa × gesneriana*. Reproduction in *Tulipa* involves both self-pollination and cross-pollination. Pollination is primarily mediated by insects, especially small flies and bees, although wind and animals may additionally contribute to pollen dispersal [52]. Natural interspecific hybridization has been reported in several *Tulipa* taxa; however, most interspecific crosses are restricted by substantial pre- and post-zygotic reproductive barriers [53,54,55,56]. Despite this, evaluating the frequency and extent of hybridization under natural conditions remains difficult. *Tulipa × tschimganica* Botschantz. is regarded as a stabilized hybrid derived from *T. kaufmanniana* and *T. dubia* Vved. [19], both of which readily hybridize with *T. greigii* [7,57]. Likewise, *T. ostrowskiana* and *T. kolpakowskiana* have been reported to produce numerous natural hybrids [4,31,53,54,55,56].

Thus, the studies described above and the limited number of studies on the morphology of the genus *Tulipa* indicate the need for a comprehensive study of the genus that incorporates morphological, anatomical, and molecular approaches.

## 3. Taxonomy and Geographic Diversity

The taxonomy of the genus *Tulipa* is complex and has been revised on numerous occasions over the past few decades. One of the most widely recognized classification systems is that proposed by Zonneveld, based on nuclear DNA data obtained by flow cytometry and morphological characteristics. Taxonomically, the genus *Tulipa* is subdivided into four subgenera (*Clusianae* (Baker) Zonn., *Eriostemones* Boiss. Raamsd., *Orithyia* (D. Don) Baker, and *Tulipa*) comprising a total of 12 sections. Based on the taxonomic treatment proposed by Zonneveld (2009), these sections include: *Clusianae* Baker (three species); *Orithyia* (D.Don) Vved. (four species); *Kolpakowskianae* Raamsd. ex Zonn. & Veldk. (16 species); *Multiflorae* (Raamsd.) Zonn. (three species); *Lanatae* (Raamsd.) Zonn. comb. et stat. nov. (eight species); *Vinistriatae* (Raamsd.) Zonn. (six species); *Spiranthera* Vved. ex Zonn. & Veldk. (four species); *Tulipanum* Reboul (11 species); *Tulipa* (nine species); *Sylvestres* (Baker) Baker (11 species); *Biflores* A.D. Hall ex Zonn. & Veldk. (15 species); and Saxatiles (Baker) Baker (six species) [7]. Among the approximately 100 recognized species of *Tulipa*, 50 have been assessed for the IUCN Red List of Threatened Species, of which 46 are endemic, highlighting the remarkable level of endemism and the conservation significance of the genus (Table 1).

**Figure 1:**
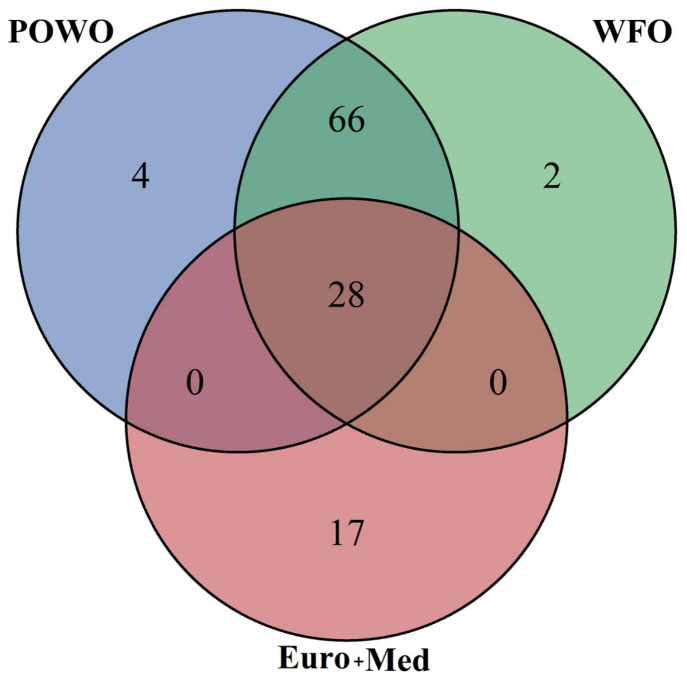
Venn diagram showing the overlap and uniqueness of Tulipa species records among three taxonomic databases: Plants of the World Online (POWO), World Flora Online (WFO), and Euro+Med PlantBase (Euro+Med). Numbers within each section indicate the number of *Tulipa* species recorded exclusively in a single database or shared among two or more databases. The central overlap (28 species) represents species common to all three databases. Unique records were represented by 4 species in POWO, 2 species in WFO, and 17 species in Euro+Med. Colored circles correspond to the respective databases: POWO (blue); WFO (green); and Euro+Med (pink).

According to the global taxonomic database published by the Royal Botanic Gardens, Kew, Plants of the World Online (POWO) [21], 98 taxa are currently accepted. Based on the World Flora Online (WFO) [22], 96 taxa of the genus *Tulipa* are currently recognized. Several taxa are listed under synonymous names, with *T. mongolica* Y.Z. Zhao treated as a synonym of *T. ulophylla* Wendelbo, and *T. tarda* Stapf—*T. urumiensis* Stapf var. *tarda* [21]. Also, *T. pseudoferganica* Lazkov and *T. sarvestanica* Alipour & Majidi are not included in the WFO and Euro+Med PlantBase databases (Euro+Med) [22,23]. As listed in the WFO database, two species (*T. praestans* Tubergen ex Hoog and *T. urumiensis*) are not listed in the POWO and Euro+Med databases [21,23]. As stated by the Euro+Med [23], 45 species of the genus *Tulipa* are currently recognized, including 19 taxa (*T. bakeri* A. D. Hall, *T. bithynica* Baker, *T. confusa* Gabrieljan, *T. cinnabarina* K. Perss. subsp. *cinnabarina*, *T. cinnabarina* subsp. *toprakii* Yıldırım & Eker, *T. eichleri* Regel, *T. florenskyi* Woronow, *T. kaghyzmanica* Fomin, *T. lownei* Baker, *T. luanica* Millaku, *T. patens*, *T. pirinica* Delip., *T. rhodopea* (Velen.) Velen., *T. schrenkii* Regel, *T. sylvestris* subsp. *australis* (Link) Pamp., *T. sylvestris* subsp. *cuspidata* (Regel) Maire & Weiller, *T. sylvestris* subsp. *primulina* (Baker) Maire & Weiller, *T. sylvestris* L. subsp. *sylvestris*, *T. urumoffii* Hayek), which are unlisted in the POWO and WFO databases, which may reflect insufficient taxonomic resolution or the need for further clarification of their systematic status (Figure 1). The Venn diagram illustrates the degree of similarity and uniqueness among species records from three taxonomic databases: POWO, WFO, and Euro+Med. The greatest overlap is observed between POWO and WFO, with 94 species shared by both databases, indicating a high level of taxonomic consistency and similarity in species coverage. However, only 28 species are represented in all three databases, suggesting limited overall agreement across platforms. POWO identified four unique species absent from both WFO and Euro+Med, whereas WFO recorded two unique species. Euro+Med contains the largest number of unique taxa, 17 species are recorded exclusively in this database (in total 45 taxa), highlighting its specific taxonomic composition and potentially greater regional specialization. There are no overlaps between POWO and Euro+Med alone, or between WFO and Euro+Med alone (no species), further confirming the differences in the data composition. Overall, the results indicate a high degree of correspondence between POWO and WFO, while Euro+Med exhibits more pronounced taxonomic isolation.

In accordance with the International Union for Conservation of Nature (IUCN) Red List of Threatened Species [24], the distribution of taxa by conservation category is as follows: one species (*T. sprengeri*) as Extinct in the Wild (EW), seven species (*T. akamasica*, *T. albanica*, *T. bactriana* J.de Groot & Tojibaev, *T. boettgeri* Regel, *T. dianaeverettiae* J.de Groot & Zonn., *T. ivasczenkoae* Epiktetov & Belyalov, *T. uzbekistanica* Botschantz. & Sharipov) are classified as critically endangered (CR), seven species (*T. cypria* Stapf ex Turrill, *T. kolbintsevii* Zonn., *T. orithyioides* Vved., *T. regelii*, *T. scharipovii* Tojibaev, *T. talassica* Lazkov, *T. toktogulica* B.D.Wilson & Lazkov) as endangered (EN), 10 species (*T. anisophylla*, *T. butkovii* Botschantz., *T. carinata* Vved., *T. fosteriana* W.Irving, *T. jacquesii* Zonn., *T. lemmersii*, *T. platystemon* Vved., *T. praestans*, *T. subquinquefolia* Vved., *T. zonneveldii* J.de Groot & Tojibaev) as vulnerable (VU), 14 species (*T. alberti*, *T. borszczowii*, *T. dubia*, *T. hungarica* Borbás, *T. iliensis* Regel, *T. ingens* Hoog, *T. kaufmanniana*, *T. kolpakowskiana*, *T. korolkowii*, *T. lanata* Regel, *T. lehmanniana*, *T. ostrowskiana*, *T. uniflora*, *T. vvedenskyi* Botschantz.) as near threatened (NT), and 13 species (*T. agenensis* Redouté, *T. altaica*, *T. bifloriformis*, *T. cretica*, *T. dasystemon*, *T. ferganica* Vved., *T. greigii*, *T. heteropetala*, *T. heterophylla* (Regel) Baker, *T. hissarica* Popov & Vved., *T. tetraphylla*, *T. turkestanica*, *T. urumiensis*) as least threatened (LC) [24]. It should be noted that, although conservation assessments are available for several species, insufficient data remain for some taxa to allow a reliable evaluation of their conservation status. In addition, a substantial number of species have not yet been assessed under the IUCN Red List criteria and therefore remain categorized as NE.

Among the accepted taxa of the genus *Tulipa*, 9 taxa are widely distributed species covering transcontinental regions, 33 species are widely distributed species in several different countries within a continent, and 79 taxa are range-restricted species with subcategories. The latter include 5 transnational endemics shared between national borderlines of neighbouring countries, 52 single-country endemics, 10 single-region endemics within national territories, and 12 single-site endemic species [21,22,23].

The main centers of species richness are located in the Tien Shan and Pamir-Alai mountain ranges. These regions are characterized by a wide range of natural conditions, including altitudes, climatic regimes, and habitat types, that support a significant number of endemic species. At the same time, many species have a transboundary distribution across several Central Asian countries, complicating assessments of their conservation status and necessitating a regional approach to the study and conservation of biodiversity [3,4,11,58].

According to POWO, the greatest diversity of *Tulipa* species is found in Central Asia and the Middle East [21]. In this region, approximately 66 species have been reported, with Kazakhstan and Uzbekistan representing important centers of *Tulipa* diversity worldwide [11]. In Kazakhstan, about 35 species have been recorded, of which 18 are listed in the Red Book of Kazakhstan and are under state protection [24].

Kazakhstan is one of the key centers of species diversity for the genus *Tulipa*, where, in accordance with current data, 31 species are recorded by POWO [12], whilst Wilson [4] lists 36 species, including a significant number (nine) of endemic species (Table 1 and Table 2). Comprehensive studies based on more than 1900 recorded locations have shown that the greatest species diversity is concentrated in the country’s southern regions. The main centers of *Tulipa* species diversity are confined to the mountainous and foothill areas of southern and southeastern Kazakhstan, including the Karatay Mountains, the Zhetysu (Dzungarian) Alatau, the Ile Alatau, and the Kungey Alatau. A high concentration of endemic taxa characterizes these areas and represents the main centers of floristic diversity [12,59]. Among the most significant species are rare and protected taxa, such as *T. alberti* and *T. greigii*, which are important components of natural ecosystems but are subject to significant anthropogenic pressure, including habitat degradation, land development, and urbanization [60]. Certain species, such as *T. tarda* [19,61], exhibit a narrow range, occurring at altitudes of 1100–1900 m in steppe and semi-steppe communities with high floristic richness. Such communities host many rare and protected species, underscoring the importance of conserving not only individual *Tulipa* populations but also entire plant communities [61]. Kazakhstan is thus one of the most important centers of diversity for the genus *Tulipa*, characterized by high species richness, significant endemism, and the marked vulnerability of wild populations, necessitating comprehensive measures for their conservation and monitoring [4,12].

In Uzbekistan, 34 *Tulipa* taxa are recognized by Tojibaev et al. [25,62]. Based on POWO, there are 31 species, whereas Wilson [4] lists 32, of which six are endemic (Table 1 and Table 2). The taxa were mapped using GIS software (ArcGIS 9.3), and their distribution was analyzed. Six species are endemic to the country. The ‘hotspots’ of *Tulipa* diversity in Uzbekistan are the western Tien Shan (18 taxa), the western Pamir–Alai Mountains (18 taxa), and the Turan Lowland (five taxa) [10,11,26]. An analysis of species distribution has shown that one of the largest centers of diversity is the Gissaro-Alai open forest ecoregion, where more than 40 *Tulipa* species are found. Additionally, the Fergana Valley plays an important role in distribution, with a high species richness and complex phytogeographical relationships [11,33]. It has been established that altitudinal zonation significantly influences *Tulipa* distribution: maximum species diversity occurs at medium altitudes, whereas at higher altitudes the number of species gradually decreases [11]. Five sections of the genus *Tulipa* are found in the Fergana Valley, representing 23 taxa (22 species) [5]. An analysis of *Tulipa* distribution in Uzbekistan showed that 27 *Tulipa* species occur in 19 protected areas. The most significant of these are the Ugam-Chatkal State National Nature Park, the Chatkal State Biosphere Reserve, and the Gissar and Surkhan State Nature Reserves, which are home to 10, nine, nine, and eight species, respectively. However, eight species (five of which are listed in the Red Data Book) occur in unprotected areas [63]. Climate was the main factor determining the distribution of most of the analyzed species; however, topography (slope angle and aspect) was equally or even more important for *T. tschimganica*, *T. borszczowii*, *T. butkovii*, *T. carinata*, *T. affinis* Botschantz., *T. undulatifolia* Boiss. (syn. *T. micheliana* Hoog), *T. biflora* (syn. *T. sogdiana* Bunge), and *T. korolkowii.* Soil was an important factor only for *T. uzbekistanica*, *T. butkovii*, and *T. lanata* Regel. The conservation implications of these results are discussed by Asatollaev et al. [10]. It has been established that *Tulipa* distribution is determined by the combined influence of climate, soil type, and topography, with the greatest species richness observed at altitudes of 700–2200 m, where optimal ecological conditions are created for most taxa [10]. In 2014, a new species, *T. intermedia* Tojibaev & J.de Groot, was discovered in Uzbekistan’s Fergana Valley; it belongs to the section *Tulipa* Kolpakowskianae Raamsd. ex Zonn. & Veldkamp. It is closely related to *T. scharipovii* and *T. talassica*. However, it differs in the morphology of its bulbs and flowers, as well as in its ecology, growing in the wormwood steppes of the plains and low foothills with gravelly slopes. Two forms of the species have been recorded, differing in the coloration of the tepals and filaments [26].

According to data from POWO [21] and Wilson [4] 28 species of the genus *Tulipa* have been recorded in Kyrgyzstan, of which six are endemic (Table 1 and Table 2). However, in recent years, a new species—*T. talassica*—has been identified from herbarium specimens and described from the Talas Range. This species is most closely related to *T. ostrowskiana*, *T. kolpakowskiana*, and *T. tianschanica* Regel, from which it differs in its strongly elongated bulb scales and erect flowers in the bud [27]. Furthermore, local pockets of morphological variability have been identified in natural populations of *T. greigii*, including differences in petal color (yellow and orange forms) [58]. *Tulipa jacquesii* is a new endemic species described in 2015 from Kyrgyzstan (western part), belonging to the section *Tulipa* sect. Biflores. White tepals distinguish it with a yellow spot, a fragrance, almost smooth tepals, two leaves, and up to four flowers. The ridges on the leaves are characteristic, bringing it closer to *T. regelii*.

Iran is one of the centers of diversity for wild *Tulipa*. As indicated in POWO [21], 24 species are recorded in the country, whereas Wilson’s study [4] notes up to 28 species, reflecting differences in taxonomic approaches. In Iran, six endemic *Tulipa* species are recorded (Table 1 and Table 2). During the study, 208 samples collected from natural areas in the provinces of Markazi and Isfahan were classified as belonging to the species *T. systola* Stapf (syn. *T. stapfii* Turril), *T. humilis* Herb, *T. biflora* (syn. *T. polychroma* Stapf), *T. biebersteiniana*, and *T. montana* Lindl. An assessment based on 44 morphological characters revealed high intraspecific and interspecific variability. The results obtained indicate the high genetic potential of the studied populations and their promise for breeding programs [64].

In Tajikistan, due to data from POWO [21] and Wilson [4], 21 and 24 species of *Tulipa* have been recorded, respectively, of which three are endemic (Table 1 and Table 2). In Afghanistan, there are 12 and 14 species, respectively, as indicated in data from POWO [21] and Wilson [4]. In China, mainly in Xinjiang region, 12 and 17 species have been recorded, respectively.

**Table 1:** Accepted species of the genus *Tulipa*.

Taxon	First Published	Native Range	Ploidy Level	References
*T. agenensis* Redouté ^×6^	1804	Cyprus, Lebanon–Syria, Palestine, Türkiye, Algeria, Corsica, Cyprus, Greece, Croatia, France, Monaco, Palestine–Jordan, Israel/Palestine, Italy, San Marino, Vatican City, Morocco, Portugal, Romania, Sardinia, Malta, Sicily, Switzerland, Tunisia	Diploid (24); Triploid (36)	[65]
*T. akamasica* Christodoulou, Hand & Charalamb. *^×2^	2014	Cyprus	Diploid (24)	[66]
*T. albanica* Kit Tan & Shuka *^×2^	2010	Albania	Diploid (24)	[67]
*T. alberti* Regel *^×5^	1877	Kazakhstan, Kyrgyzstan	Diploid (24)	[68]
*T. aleppensis* Boiss. ex Regel ^×7^	1873	Lebanon–Syria, Türkiye	Diploid (24); Triploid (36)	[19]
*T. altaica* Pall. ex Spreng. ^×6^	1825	Altay, Kazakhstan, West Siberia, Xinjiang	Diploid (24); Tetraploid (48)	[69]
*T. anisophylla* Vved. ^×4^	1935	Tajikistan, Uzbekistan	Diploid (24)	[70]
*T. annae* J.de Groot & Zonn. *^×8^	2020	Kazakhstan	Diploid (24)	[21]
*T. armena* Boiss. ^×8^	1859	Iran, Transcaucasus (Armenia, Georgia, Azerbaijan), Türkiye	Diploid (24); Triploid (36)	[19]
*T. auliekolica* Perezhogin *^×8^	2014	Kazakhstan	Unknown	[71]
*T. bactriana* J.de Groot & Tojibaev *^×2^	2020	Uzbekistan	Diploid (24)	[72]
*T. bakeri* A. D. Hall *^×8^	1938	Greece	Diploid (24)	[23,73]
*T. banuensis* Grey-Wilson *^×8^	1974	Afghanistan	Diploid (24)	[19]
*T. biflora* Pall. ^×8^	1776	Afghanistan, East European Russia, Egypt, Iran, Iraq, Armenia, Azerbaijan, Kazakhstan, Kyrgyzstan, Krym, Lebanon–Syria, Egypt, North Caucasus, NW. Balkan Pen. (Bosnia and Herzegovina, Croatia, Montenegro, North Macedonia, Serbia, Slovenia, Kosovo), North Macedonia, Pakistan, Palestine, Israel–Palestine, Saudi Arabia, Sinai, South European Russia, Ukraine, Tajikistan, Transcaucasus (Armenia, Georgia, Azerbaijan), Turkmenistan, Türkiye, Uzbekistan, West Siberia, Xinjiang	Diploid (24;) Triploid (36); Tetraploid (48)	[7]
*T. bifloriformis* Vved. ^×6^	1971	Kazakhstan, Kyrgyzstan, Tajikistan, Uzbekistan	Diploid (24); Triploid (36); Tetraploid (48)	[74]
*T. bithynica* Baker *^×8^	1874	Greece	Unknown	[23,73]
*T. boettgeri* Regel *^×2^	1887	Tajikistan	Unknown	[75]
*T. borszczowii* Regel ^×5^	1868	Kazakhstan, Uzbekistan	Diploid (24)	[76]
*T. botschantzevae* S.N. Abramova & Zakal. ^×8^	1973	Iran, Turkmenistan	Unknown	[19]
*T. brinkii* J.de Groot & Zonn. *^×8^	2022	Iran	Diploid (24)	[21]
*T. butkovii* Botschantz. *^×4^	1961	Uzbekistan	Diploid (24)	[77]
*T. carinata* Vved. ^×4^	1971	Afghanistan, Tajikistan, Uzbekistan	Diploid (24)	[78]
*T. cinnabarina* K. Perss. *^×8^	2000	Türkiye	Diploid (24)	[19]
*T. cinnabarina* K. Perss. subsp. cinnabarina *^×8^	2022	Türkiye	Unknown	[23]
*T. cinnabarina* subsp. *toprakii* Yıldırım & Eker *^×8^	2016	Türkiye	Unknown	[23]
*T. clusiana* Redouté ^×8^	1803	Greece, France, Monaco, Germany, Italy, San Marino, Vatican City, Portugal, Romania, Spain, Andorra, Tunisia, Türkiye, Afghanistan, Iran, Iraq, Pakistan, West Himalaya	Diploid (24); Triploid (36); Tetraploid (48); Pentaploid (60)	[19]
*T. confusa* Gabrieljan ^×8^	1966	Caucasia, Armenia, Azerbaijan	Unknown	[23]
*T. cretica* Boiss. & Heldr. *^×6^	1854	Greece (Kriti)	Diploid (24)	[79]
*T. cypria* Stapf ex Turrill *^×3^	1934	Cyprus	Triploid (36)	[80]
*T. dasystemon* (Regel) Regel ^×6^	1879	Kazakhstan, Kyrgyzstan, Tajikistan, Uzbekistan, Xinjiang	Diploid (24); Tetraploid (48)	[81]
*T. dianaeverettiae* J.de Groot & Zonn. *^×2^	2020	Kazakhstan	Tetraploid (48)	[82]
*T. dubia* Vved. ^×5^	1935	Kazakhstan, Kyrgyzstan, Uzbekistan	Diploid (24)	[83]
*T. eichleri* Regel ^×8^	1874	Caucasia, Azerbaijan, Georgia	Unknown	[23]
*T. ferganica* Vved. ^×6^	1935	Kyrgyzstan, Uzbekistan	Diploid (24)	[84]
*T. florenskyi* Woronow ^×8^	1924	Caucasia, Armenia, Azerbaijan	Unknown	[23]
*T. foliosa* Stapf *^×8^	1885	Türkiye	Unknown	[19]
*T. fosteriana* W.Irving ^×4^	1906	Afghanistan, Kyrgyzstan, Tajikistan, Uzbekistan	Diploid (24)	[85]
*T. gesneriana* L. ^×8^	1753	Türkiye, Albania, Baltic states, Belarus, Finland, Czech Republic, Slovakia, France, Monaco, Germany, Great Britain, Greece, Israel/Palestine–Jordan, Italy, San Marino, Vatican City, Lebanon–Syria, Moldova, Romania, Russia; Spain, Andorra, Sweden, Switzerland, Ukraine	Diploid (24)	[19,86,87]
*T. greigii* Regel ^×6^	1873	Iran, Kazakhstan, Kyrgyzstan, Tajikistan, Uzbekistan	Diploid (24)	[88]
*T. harazensis* Rech.f. *^×8^	1990	Iran	Unknown	[19]
*T. heteropetala* Ledeb. ^×6^	1829	Altay, Kazakhstan, Xinjiang	Diploid (24)	[89]
*T. heterophylla* (Regel) Baker ^×6^	1874	Kazakhstan, Kyrgyzstan, Xinjiang	Diploid (24)	[90]
*T. heweri* Raamsd. *^×8^	1998	Afghanistan	Diploid (24)	[19]
*T. hissarica* Popov & Vved. ^×6^	1935	Tajikistan, Uzbekistan	Diploid (24)	[91]
*T. hoogiana* B.Fedtsch. ^×8^	1910	Iran, Turkmenistan	Diploid (24); Triploid (36)	[19]
*T. humilis* Herb. ^×8^	1844	Afghanistan, Iran, Iraq, Lebanon–Syria, Russia (North Caucasus), Transcaucasus (Armenia, Georgia, Azerbaijan), Türkiye	Diploid (24)	[19]
*T. hungarica* Borbás ^×5^	1882	Bulgaria, Greece, Serbia, Montenegro, Kosovo, Romania	Diploid (24)	[92]
*T. iliensis* Regel ^×5^	1879	Kazakhstan, Kyrgyzstan, Xinjiang	Diploid (24); Triploid (36)	[93]
*T. ingens* Hoog ^×5^	1902	Tajikistan, Uzbekistan	Diploid (24)	[94]
*T. intermedia* Tojibaev & J.de Groot *^×8^	2014	Uzbekistan	Unknown	[71,95]
*T. ivasczenkoae* Epiktetov & Belyalov *^×2^	2013	Kazakhstan	Unknown	[96]
*T. jacquesii* Zonn. *^×4^	2015	Kyrgyzstan	Diploid (24)	[97]
*T. julia* K.Koch ^×8^	1849	Lebanon–Syria, Transcaucasus (Armenia, Georgia, Azerbaijan), Türkiye	Diploid (24)	[19]
*T. kaghyzmanica* Fomin ^×8^	1908	Caucasia, Armenia, Türkiye	Unknown	[23]
*T. kaufmanniana* Regel ^×5^	1877	Kazakhstan, Kyrgyzstan, Tajikistan, Uzbekistan	Diploid (24); Triploid (36)	[98]
*T. kolbintsevii* Zonn. *^×3^	2012	Kazakhstan	Diploid (24)	[99]
*T. kolpakowskiana* Regel ^×5^	1877	Afghanistan, Kazakhstan, Kyrgyzstan, Xinjiang	Diploid (24); Tetraploid (48)	[100]
*T. korolkowii* Regel ^×5^	1875	Kazakhstan, Kyrgyzstan, Tajikistan, Uzbekistan	Diploid (24)	[101]
*T. kosovarica* Kit Tan, Shuka & Krasniqi ^×8^	2012	Albania, NW. Balkan Pen. (Serbia, Kosovo, Montenegro)	Unknown	[19]
*T. koyuncui* Eker & Babaç *^×8^	2010	Türkiye	Diploid (24)	[19]
*T. kuschkensis* B.Fedtsch. ^×8^	1932	Afghanistan, Iran, Turkmenistan	Diploid (24)	[19]
*T. lanata* Regel ^×5^	1884	Afghanistan, Pakistan, Tajikistan, Uzbekistan, West Himalaya	Diploid (24); Triploid (36)	[102]
*T. lehmanniana* Merckl. ^×5^	1852	Afghanistan, Iran, Kazakhstan, Kyrgyzstan, Tajikistan, Turkmenistan, Uzbekistan	Diploid (24); Triploid (36)	[103]
*T. lemmersii* Zonn., Peterse & J.de Groot *^×4^	2012	Kazakhstan	Diploid (24)	[104]
*T. linifolia* Regel ^×8^	1884	Afghanistan, Iran, Tajikistan, Uzbekistan	Diploid (24)	[19]
*T. lorestanica* Rukšāns & Zubov *^×8^	2022	Iran	Unknown	[12,105]
*T. lownei* Baker ^×8^	1874	Lebanon–Syria	Unknown	[23]
*T. luanica* Millaku *^×8^	2015	NW. Balkan Pen. (Kosova, Serbia, Montenegro)	Diploid (24)	[71,106]
*T. mongolica* Y.Z.Zhao *^×8^	2003	Inner Mongolia (China)	Unknown	[107]
*T. montana* Lindl. ^×8^	1827	Iran, Turkmenistan	Diploid (24)	[7,21]
*T. narcissicum* N.Y.Stepanova *^×8^	2014	South European Russia	Unknown	[71]
*T. orithyioides* Vved. ^×3^	1935	Kyrgyzstan, Tajikistan, Uzbekistan	Diploid (24)	[108]
*T. orphanidea* Boiss. ex Heldr. ^×8^	1862	Bulgaria, East Aegean Is., Greece, Kriti, Türkiye, Türkiye-in-Europe	Diploid (24); Triploid (36) Tetraploid (48)	[19]
*T. ostrowskiana* Regel ^×5^	1884	Kazakhstan, Kyrgyzstan	Tetraploid (48)	[109]
T. patens * ^×8^	1829	Russia	Diploid (24)	[23,110]
*T. persica* (Lindl.) Sweet *^×8^	1830	Iran	Diploid (24)	[19]
*T. pirinica* Delip. *^×8^	1987	Bulgaria	Unknown	[23]
*T. platystemon* Vved. *^×4^	1935	Kyrgyzstan	Unknown	[111]
*T. praestans* H.B.May *^×4^	1903	Tajikistan	Diploid (24)	[112]
*T. pseudoferganica* Lazkov *^×8^	2021	Kyrgyzstan	Diploid (24)	[21]
*T. regelii* Krasn. *^×3^	1888	Kazakhstan	Diploid (24)	[113]
*T. rhodopea* (Velen.) Velen. *^×8^	1995	Bulgaria	Unknown	[23]
*T. salsola* Rukšāns & Zubov *^×8^	2022	Kazakhstan	Unknown	[21]
*T. sarvestanica* Alipour & Majidi *^×8^	2023	Iran	Unknown	[21,114]
*T. saxatilis* Sieber ex Spreng. ^×8^	1825	Greece (East Aegean Is., Kriti), Italy, San Marino, Vatican City, Türkiye	Diploid (24); Triploid (36)	[19]
*T. scardica* Bornm. ^×8^	1923	NW. Balkan Pen. (North Macedonia, Serbia, Kosovo, Montenegro, Greece)	Diploid (24)	[115]
*T. schachimardanica* Khalk. *^×8^	1984	Uzbekistan	Unknown	[21]
*T. scharipovii* Tojibaev *^×3^	2009	Kyrgyzstan, Uzbekistan *	Unknown	[116]
*T. schmidtii* Fomin ^×8^	1909	Iran, Transcaucasus (Armenia, Georgia, Azerbaijan), Azerbaijan	Diploid (24)	[19]
*T. schrenkii* Regel ^×8^	1873	Russia, Ukraine, Kazakhstan	Diploid (24)	[23]
*T. serbica* Tatic & Krivošej ^×8^	1997	NW. Balkan Pen. (Serbia, Kosovo, Montenegro)	Unknown	[19]
*T. sinkiangensis* Z.M.Mao *^×8^	1980	Xinjiang (China)	Diploid (24)	[19]
*T. sosnowskyi* Achv. & Mirzoeva *^×8^	1950	Transcaucasus (Armenia, Azerbaijan)	Diploid (24)	[19]
*T. sprengeri* Baker *^×1^	1894	Türkiye	Diploid (24)	[19]
*T. suaveolens* Roth ^×8^	1794	Kazakhstan, Krym, North Caucasus, Transcaucasus (Armenia, Georgia, Azerbaijan), Bulgaria, Romania	Diploid (24)	[19]
*T. subquinquefolia* Vved. *^×4^	1946	Tajikistan *, Uzbekistan	Unknown	[117]
*T. sylvestris* L. ^×8^	1753	Algeria, Austria, Liechtenstein, Estonia, Latvia, Lithuania, Belgium, Luxembourg, Denmark, Finland, Czech Republic, Slovakia, North Macedonia, Monaco, Germany, Great Britain, Hungary, Ireland, Italy, San Marino, Vatican City, Moldova, Netherlands, Norway, Poland, Russia (Central European Russia, East European Russia, South European Russia), Malta, Andorra, Sweden, Altay, Belarus, France, Greece, Iran, Kazakhstan, Krym, Libya, Morocco, North Caucasus, NW. Balkan Pen. (Albania, Bosnia-Herzegovina, Bulgaria, Kosovo, Montenegro, Greece, Serbia, Croatia), Portugal, Romania, Sardinia, Sicilia, Spain, Switzerland, Transcaucasus (Armenia, Georgia, Azerbaijan), Tunisia, Türkiye, Ukraine, West Siberia, Xinjiang	Diploid (24), Triploid (36), Tetraploid (48)	[19]
*T. sylvestris* subsp. *australis* (Link) Pamp. ^×8^	1914	Albania, Algeria, Bulgaria, Caucasia, Azerbaijan, Georgia, Bosnia-Herzegovina, Croatia, North Macedonia, Serbia, Kosovo, Montenegro, France, Monaco, Greece, Italy, San Marino, Vatican City, Libya, Moldova, Morocco, Portugal, Romania, Russia, Sardinia, Sicily, Malta, Spain, Gibraltar, Andorra, Andorra, Switzerland, Tunisia, Türkiye, Ukraine	Diploid (24)	[23]
*T. sylvestris* subsp. *cuspidata* (Regel) Maire & Weiller *^×8^	1884	Algeria	Unknown	[23]
*T. sylvestris* subsp. *primulina* (Baker) Maire & Weiller ^×8^	1882	Algeria, Morocco	Diploid (24)	[23,118]
*T. sylvestris* L. subsp. *sylvestris* ^×8^	1829	Albania, Algeria, Austria, Liechtenstein, Belgium, Luxembourg, Bulgaria, Denmark, Czech Republic, Bosnia-Herzegovina, Croatia, France, Monaco, Germany, Great Britain, Greece, Hungary, Italy, San Marino, Vatican City, Morocco, Netherlands, Norway, Poland, Romania, Sardinia, Sicily, Malta, Spain, with Gibraltar, Andorra, Sweden, Switzerland, Türkiye, Ukraine	Diploid (24); Triploid (36)	[23]
*T. systola* Stapf ^×8^	1885	Iran, Iraq, Israel/Palestine–Jordan, Lebanon–Syria, Palestine, Sinai, Türkiye	Diploid (24)	[19]
*T. talassica* Lazkov *^×3^	2011	Kyrgyzstan *, Uzbekistan	Unknown	[20,119]
*T. tarda* Stapf ^×8^	1933	Iran, Kazakhstan, Kyrgyzstan	Unknown	[19,61]
*T. tetraphylla* Regel ^×6^	1875	Kazakhstan, Kyrgyzstan, Xinjiang	Diploid (24); Tetraploid (48)	[120]
*T. toktogulica* B.D.Wilson & Lazkov *^×3^	2022	Kyrgyzstan	Unknown	[20,121]
*T. × tschimganica* Botschantz. *^×8^	1961	Kyrgyzstan, Uzbekistan *	Diploid (24)	[19]
*T. turgaica* Perezhogin *^×8^	2014	Kazakhstan	Unknown	[61,122]
*T. turkestanica* (Regel) Regel ^×6^	1875	Kyrgyzstan, Tajikistan, Uzbekistan, Xinjiang, Germany	Diploid (24); Tetraploid (48)	[123]
*T. ulophylla* Wendelbo *^×8^	1967	Iran	Diploid (24)	[71]
*T. undulatifolia* Boiss. ^×8^	1844	East Aegean Is., Greece, Iran, North Caucasus, NW. Balkan Pen. (North Macedonia, Serbia, Kosovo, Montenegro), Romania, Tajikistan, Transcaucasus (Armenia, Georgia, Azerbaijan), Turkmenistan, Türkiye, Türkiye-in-Europe, Uzbekistan	Diploid (24); Triploid (36)	[124]
*T. uniflora* (L.) Besser ex Baker ^×5^	1874	Altay, Inner Mongolia, Irkutsk, Kazakhstan, Krasnoyarsk, Mongolia, Tuva, Xinjiang	Diploid (24)	[125]
*T. uzbekistanica* Botschantz. & Sharipov *^×2^	1971	Uzbekistan	Unknown	[62,126]
*T. vvedenskyi* Botschantz. ^×5^	1954	Tajikistan, Uzbekistan	Diploid (24); Triploid (36)	[127]
*T. zonneveldii* J.de Groot & Tojibaev *^×4^	2017	Kyrgyzstan	Unknown	[128,129]
*T. praestans* Tubergen ex Hoog *^×8^	1903	Tajikistan	Diploid (24)	[130]
*T. urumiensis* Stapf var. tarda ^×6^	2017	Iran, Kazakhstan, Kyrgyzstan	Diploid (24)	[131]
*T. urumoffii* Hayek ^×8^	1911	Bulgaria, Romania	Diploid (24)	[23]

*—indicates an endemic species. ^×^—indicates species included in the IUCN Red List of Threatened Species: ^×1^—Extinct in the Wild (EW); ^×2^—Critically Endangered (CE); ^×3^—Endangered (EN); ^×4^—Vulnerable (VU); ^×5^—Near Threatened (NT); ^×6^—Least Concern (LC); ^×7^—Data Deficient (DD); ^×8^—Not Evaluated (NE).

**Table 2:** Range-Distribution Categories of *Tulipa*.

Distribution Category	Representative *Tulipa* Taxa
Widely distributed species covering transcontinental regions	*T. agenensis*, *T. biflora*, *T. clusiana*, *T. gesneriana*, *T. sylvestris*, *T. sylvestris* subsp. *australis* (Link) Pamp., *T. sylvestris* L. subsp. *sylvestris*, *T. suaveolens*, *T. undulatifolia*
Widely distributed species in several countries within one continent	*T. alberti*, *T. anisophylla*, *T. bifloriformis*, *T. borszczowii*, *T. dubia*, *T. hissarica*, *T. ingens*, *T. kaufmanniana*, *T. korolkowii*, *T. orithyioides*, *T. ostrowskiana*, *T. scharipovii*, *T. subquinquefolia*, *T. talassica*, *T. × tschimganica*, *T. vvedenskyi* (Central Asia); *T. aleppensis*, *T. armena*, *T. hungarica*, *T. kosovarica*, *T. orphanidea*, *T. scardica*, *T. serbica*, *T. luanica*, *T. urumoffii* (Europe); *T. julia, T. schmidtii*, *T. sosnowskyi*, *T. systola*, *T. confusa*, *T. eichleri*, *T. florenskyi*, *T. kaghyzmanica* (Western Asia (Middle East)).
Range-restricted species. Species with subcategories:	
Transnational endemics	*T. alberti* (Kazakhstan–Kyrgyzstan); *T. scharipovii* (Kyrgyzstan–Uzbekistan); *T. sosnowskyi* (Transcaucasus-South Caucasus); *T. subquinquefolia* Vved. (Tajikistan-Uzbekistan); *T. talassica* (Kyrgyzstan–Uzbekistan).
Single-country endemics	*T. annae*, *T. auliekolica*, *T. dianaeverettiae*, *T. ivasczenkoae*, *T. kolbintsevii*, *T. lemmersii*, *T. regelii*, *T. salsola*, *T. turgaica* (Kazakhstan); *T. bactriana*, *T. butkovii*, *T. intermedia*, *T. schachimardanica*, *T. × tschimganica*, *T. uzbekistanica* (Uzbekistan); *T. jacquesii*, *T. platystemon*, *T. pseudoferganica*, *T. talassica*, *T. toktogulica*, *T. zonneveldii* (Kyrgyzstan); *T. brinkii*, *T. harazensis*, *T. lorestanica, T. persica*, *T. sarvestanica*, *T. ulophylla* (Iran); *T. cinnabarina*, *T. foliosa*, *T. koyuncui*, *T. sprengeri*, *T. cinnabarina* K. Perss. subsp. *cinnabarina*, *T. cinnabarina* subsp. *toprakii* Yıldırım & Eker (Türkiye); *T. boettgeri*, *T. praestans*, *T. subquinquefolia* (Tajikistan); *T. bakeri*, *T. bithynica*, *T. cretica*, (Greece); *T. banuensis*, *T. heweri* (Afghanistan); *T. mongolica*, *T. sinkiangensis* (Inner Mongolia and Xinjiang, China); *T. akamasica*, *T. cypria* (Cyprus); *T. pirinica*, *T. rhodopea* (Bulgaria); *T. narcissicum*, *T. patens* (Russia); *T. albanica* (Albania); *T. luanica* (NW. Balkan Pen., Kosovo); *T. sylvestris* subsp. *cuspidata* (Regel) Maire & Weiller (Algeria).
Single-region endemics within national territories	*T. auliekolica* (Northern Kazakhstan, Kazakhstan); *T. cretica* (Crete/Kriti, Greece); *T. bithynica* (East Aegean islands, Greece); *T. sinkiangensis* (Xinjiang, China); *T. mongolica* (Inner Mongolia region, China); *T. narcissicum* (South European Russia region, Russia); *T. patens* (European Russia, Russia); *T. luanica* (NW Balkan Peninsula); *T. pirinica* (Pirin region, Bulgaria); *T. rhodopea* (Rhodope Mountains, Bulgaria).
Single-site endemics	*T. annae* (Taskora Gorge, Dzungarian Alatau, Kazakhstan); *T. lemmersii* (Mashad Pass, Kazakhstan); *T. dianaeverettiae* (Altai Pass, Kazakhstan); *T. ivasczenkoae* (mts. Chulak, Dzhungarian Alatau, Kazahstan); *T. akamasica* (Akamas Peninsula, Cyprus); *T. albanica* (Surroj, Albania); *T. bactriana* (South-Western Pamir-Alay: Sherabad Valley, Uzbekistan); *T. uzbekistanica* (Gissar Range, Uzbekistan); *T. bakeri, T. cretica* (Kriti Island, Greece); *T. toktogulica* (Toktogul area, Kyrgyzstan); *T. pseudoferganica* (Sary-Chelek Reserve, Chatkal Range, Kyrgyzstan).

Based on POWO (Plants of the World Online), seven species of the genus *Tulipa* have been recorded in Turkmenistan, whereas 18 species, two subspecies, and two varieties have been identified in the flora of Türkiye [21]. In Türkiye, six endemic *Tulipa* taxa are recorded (Table 1 and Table 2). These data are based on an analysis of herbarium and field specimens, which has enabled clarification of the taxonomic status of several taxa and confirmation of previously doubtful species [132], including *T. sprengeri*, which is considered extinct. At the same time, Wilson’s work notes 17 species of *Tulipa* in the region, reflecting differences in taxonomic interpretation and nomenclature updates. *Tulipa orphanidea* Boiss. ex Heldr. is distributed in the Eastern Mediterranean, mainly in the Balkans and Turkey. In the western part of the range (Manisa province), isolated populations exhibiting genetic differentiation have been recorded [133].

The Balkan region, and particularly Greece, represents an important secondary centre of tulip diversity. As indicated in Vascular Flora of Greece (Vascular Flora of Greece), 11 species of *Tulipa* occur in Greece, of which nine are native (*T. australis*, *T. bakeri*, *T. clusiana* Redouté, *T. cretica* Boiss. & Heldr., *T. orphanidea*, *T. rhodopea*, *T. saxatilis* Sieber ex Spreng., *T. scardica T. undulatifolia*), while two (*T. agenensis* Redouté, *T. bithynica*) are considered fully naturalized, having escaped from cultivation in the past [29,134]. Some of these species are single-country endemics (*T. bakeri*, *T. bithynica*, *T. cretica*) and listed (*T. agenensis*, *T. cretica*) in the IUCN Red List of Threatened Species but also remain subject to ongoing taxonomic uncertainty and revision (Table 1 and Table 2) [65,79]. Eight species have been recorded in the POWO database in Greece, while seven taxa of the genus *Tulipa* have been recorded in the flora of Northwest Balkan Peninsula (*T. biflora*, *T. kosovarica* Kit Tan, Shuka & Krasniqi, *T. luanica* Millaku, *T. scardica*, *T. serbica*, *T. sylvestris*, *T. undulatifolia*) [19,71,83,106]. Within the Deva serpentinite massif, the sympatric coexistence of endemic and widespread taxa has been identified, indicating a high level of taxonomic diversity within a limited area. This area is considered an important local center of *Tulipa* diversity in the Western Balkans [21,135]. *Tulipa* species in the Balkans exhibit complex endemism and close phylogenetic relationships. *Tulipa serbica* from Serbia was previously considered a form of *T. scardica* [136]. *Tulipa kosovarica* and *T. albanica* Kit Tan & Shuka are endemic to serpentinite substrates in Kosovo and Albania and are phylogenetically close to *T. scardica* and *T. schrenkii* [48,137]. *Tulipa luanica* is described from limestone substrates in southern Balkans and forms part of a complex of closely related taxa (*T. australis*, *T. gesneriana*, *T. kosovarica*, *T. serbica*, and *T. albanica*) [106]. Overall, the group shows high taxonomic complexity and ecological differentiation in the Balkans.

In China and Afghanistan, 13 and 12 species have been recorded, respectively. In Russia, including the Altai and Siberian regions, nine species are recorded, and the same number is reported in the Caucasus regions. China (Inner Mongolia, Xinjiang) and Afghanistan each have two endemic *Tulipa* species (Table 1 and Table 2) [21].

Previously, three species were recorded in Mongolia [107]: *T. heteropetala*, *T. uniflora*, and *T. mongolica* Y.Z.Zhao. Morphological and molecular-genetic analyses confirm that two species from the subgenus *Orithyia* grow in the study area: *T. uniflora* and *T. mongolica* [21,107]. However, in similar studies by Wilson, two species were recorded: *T. dianaeverettiae* Zonn & J. de Groot and *T. uniflora* [4]. Three species each have been recorded in Albania, Ukraine, Cyprus, Pakistan, and Palestine. Two species each have been registered in Egypt and Bulgaria, respectively. Six species have been recorded in the POWO database in Lebanon–Syria, and four in Iraq (Table 1 and Table 2). Only *T. biflora* has been recorded in Saudi Arabia [21].

The genus *Tulipa* is thus characterized by a high degree of taxonomic complexity, significant intraspecific variation, and repeated revisions of its classification. The primary center of its diversity is concentrated in the mountainous regions of Central Asia (the Tien Shan and Pamir-Alai), whilst secondary centers are found in South-West Asia, the Eastern Mediterranean, and the Balkans. Across all the countries under consideration, differences are observed in the assessment of species richness among the sources: POWO [21], World Flora Online [22], Euro+Med PlantBase [23], and Wilson [4], reflecting taxonomicinstability and differences in approaches to species interpretation.

## 4. Population Genetic Structure and Molecular Diversity

In addition to taxonomic and floristic studies, research into the genetic diversity and phylogenetic relationships of species within the genus *Tulipa* has been actively developing in recent years [138,139]. Morphological analysis, based on the characteristics of bulbs, leaves, stems, and flowers, generally confirms existing classification systems, although unresolved issues remain for some taxa [4,21,31,33]. Research into the genetic diversity, systematics, and evolutionary relationships of *Tulipa* species is advancing through the application of cytogenetic and molecular genetic methods. One key approach is estimating nuclear genome size, which can serve as an important taxonomic and evolutionary marker, and using modern molecular methods that analyze various DNA markers to provide a more accurate assessment of genetic diversity and evolutionary relationships within the genus.

The size of the nuclear genome in members of the genus *Tulipa* was investigated using flow cytometric measurement of nuclear DNA. Previous studies have shown that over 400 samples representing 123 taxa, predominantly from wild populations, were analyzed. Based on a comprehensive analysis incorporating data on genome size, morphological characteristics, geographical distribution, and molecular traits, an updated taxonomy of the genus comprising 87 species was proposed [7]. In a similar study, this method was used to describe a new species, *T. kolbintsevii* Zonn. [140]. Cytological studies revealed a chromosome number of 2*n* = 2x = 24 for *T. albanica* and a genome size of 54.15 ± 0.23 pg; furthermore, two morphotypes of *T. australis* have been recorded in Albania, associated with limestone and serpentinite substrates [137]. In 2015, a new species, *T. jacquesii* was discovered in Kyrgyzstan. Its genome size is 51.9 pg, which falls within the range of variation (51.5–59.4 pg) [141]. Similarly, the species *T. luanica* is diploid (2n = 2x = 24), with a karyotype comprising 2 metacentric, 2 submetacentric, and 8 subtelocentric chromosome pairs [106]. *Tulipa pulchella* has x = 12; whilst diploid forms (2n = 2x = 24) predominate in the genus *Tulipa*, triploid, tetraploid, pentaploid, and hexaploid cytotypes are also found [142]. The karyotypes of *T. sinkiangensis* Z.M.Mao and *T. schrenkii* were analyzed based on chromosome sizes and physical mapping of 5S and 45S rDNA using fluorescence in situ hybridization. Both species were found to have the same karyotype formula: 2n = 2x = 24 = 8 sm + 16 st (four pairs of submedian and eight pairs of subterminal chromosomes), indicating their close relationship and suggesting genomic modifications during diversification [143]. Cytogenetic studies of *Tulipa* have contributed to a refined taxonomic framework for the genus. Most species exhibit a conserved chromosome number (2n = 2x = 24) alongside substantial variation in nuclear genome size, indicating high genomic plasticity. Several taxa (*T. albanica*, *T. luanica*, *T. sinkiangensis*, *T. schrenkii*) maintain a stable diploid level, although differences in karyotypes and rDNA profiles suggest cryptic genomic differentiation and close phylogenetic relationships (Table 3). Overall, diversity in *Tulipa* is driven by a combination of conserved chromosome number, variation in genome size, karyotypic changes, and polyploidy, which jointly contribute to its taxonomic and evolutionary diversity. In another study, esterase polymorphism was investigated to determine genetic relationships. A comparison of isoenzyme analysis results with morphological data showed that molecular markers can provide clearer species differentiation [144]. Due to esterase profiles, species of section *Tulipa* formed a well-defined cluster, whereas in section *Biflores* the esterase patterns exhibited high variability and did not support clear clustering, limiting their utility for assessing interspecific relationships within this group. The lack of species clustering in the results of the cluster analysis based on esterase spectra is likely due to the inability to correctly establish homology of co-migrating bands because of the high complexity of the electrophoretic patterns. Overall, this method demonstrates limited informative value, particularly when analyzing taxa at higher taxonomic levels.

In 2008, four wild *Tulipa* species and 10 cultivars from Xinjiang, China, were analyzed using random amplified polymorphic DNA (RAPD) [35]. This study showed that wild species are genetically distant from cultivars and exhibit much higher levels of genetic polymorphism. However, RAPD markers do not always provide sufficient discriminatory power to reliably assess genetic diversity.

The interspace short repetitive sequences (ISSR) method is also widely used to study the genetic structure of populations in the eastern regions of Iran. It has enabled the identification of 97 polymorphic fragments. Cluster analysis divided the samples into nine groups corresponding to seven species [36]. Similar studies were conducted on 18 natural populations of *T. × gesneriana*. from the north of the Lower Volga region and 31 natural populations of *T. suaveolens* located in the north and north-east of its range in European Russia [52,145]. Additionally, molecular genetic studies were conducted within the species *T. suaveolens* Roth using nuclear (ITS) and plastid (cpDNA) sequences [146]. These researchers analyzed 22 natural *T. suaveolens* localities in the Astrakhan, Volgograd, Orenburg, Rostov, Samara, and Saratov provinces, the Krasnodar region, the Republics of Kalmykia and Dagestan, and Crimea and western Kazakhstan [145]. These studies demonstrate that ISSR markers, ITS, and cpDNA are effective tools for studying the genetic diversity of closely related species and population variability. ISSR markers, ITS, and cpDNA are widely used as complementary tools for assessing genetic diversity and population structure in the genus *Tulipa*. However, ISSR markers are dominant and may suffer from reproducibility and amplicon homology issues, limiting their utility in detailed population genetic analyses. In contrast, ITS and cpDNA markers often exhibit insufficient variability for resolving closely related species, intraspecific taxa, and recently diverged populations. Therefore, the combined application of these marker systems provides a more robust framework for comprehensive genetic analyses in *Tulipa* [36,146].

In 2001, Amplified Fragment Length Polymorphism (AFLP) markers were used for the first time to study intraspecific variation in *T. sprengeri*. Subsequently, AFLP enabled the assessment of genetic diversity in populations of four species (*T. biebersteiniana*, *T. patens*, *T. scytica* Klokov & Zoz (syn. *T. sylvestris* subsp. *australis* (Link) Pamp.), and *T. riparia* Knjaz., Kulikov & E.G.Philippov (syn. *T. sylvestris* subsp. *australis* (Link) Pamp.) from various regions of Russia [37]. AFLP analysis was also used to assess the genetic diversity of nine *Tulipa* species in Iran, suggesting the possible establishment of a new subgenus for *T. biebersteiniana* [147]. Recently, Haerinasab et al. [148] used Conserved DNA-derived polymorphism (CDDP) for the first time in Iran to study genetic diversity and population structure. Although CDDP markers did not fully enable differentiation or unambiguous assignment of individual plants to the populations of each species, the results contribute to expanding knowledge of the genetic relationships between them [86]. AFLP markers have been widely used to assess genetic diversity and population structure in several *Tulipa* species from Russia and Iran, demonstrating their utility for detecting intraspecific polymorphism and phylogenetic relationships. More recently, CDDP markers have been used in Iranian *Tulipa* species, providing additional information on genetic relationships; however, their resolution at the population level is limited, and they are generally unable to reliably discriminate between closely related species [36,147,148]. Overall, the use of AFLP and CDDP markers demonstrates that the identified polymorphisms are informative for assessing intraspecific genetic variability in natural populations.

Recent integrative studies using the Inter Primer Binding Site (iPBS) method have revealed high genetic diversity among *Tulipa* species from Turkey and Kazakhstan and identified several genetically distinct clusters corresponding to their geographical origins, indicating a complex evolutionary history of the genus [38]. Overall, iPBS has proven highly effective in assessing genetic diversity and population structure in *Tulipa* species. This marker-based approach allows for the detection of significant levels of polymorphism and the formation of clearly differentiated genetic clusters. However, like other dominant markers, its resolution may be limited when analyzing subtle intrapopulation variation. This approach has certain limitations, such as the lack of a comparison of iPBS performance with previously used genetic analysis methods. Future studies could include such a comparison and test the validity of the iPBS approach. Thus, iPBS is an effective tool for macrogeographical and interpopulation studies, especially when combined with other molecular approaches [38].

SRAP-PCR was also performed on genetic material from 40 *Tulipa* varieties, yielding 245 polymorphic bands. Analysis of the polymorphic bands, genetic diversity indices, a cluster dendrogram, principal coordinate analysis, and a correlation heat map revealed the genetic diversity of 40 *Tulipa* varieties and genetic resources [39]. These results demonstrate that SRAP accurately reflects genetic differences in *Tulipa*. Overall, the results demonstrate that SRAP is a reliable and informative marker system for assessing genetic variation in *Tulipa* germplasm. However, as a dominant marker system, SRAP cannot distinguish between homozygous and heterozygous genotypes, which reduces the accuracy of population genetic estimates, and it may also have limited resolving power for very closely related species [39].

Large-scale transcriptome assemblies have been developed for *Tulipa* and related species, comprising tens of thousands of contigs and providing partial transcriptome coverage (approximately 37–39%). Analysis of SSRs and SNPs revealed distinct repeat distributions in coding and non-coding regions, enabling the creation of high-throughput genotyping markers. A comparative analysis of orthologous groups among *Lilium* L., and *Tulipa*, revealed conserved genetic relationships and facilitated the identification of common SNP and SSR markers for studying synteny and gene functional annotation [40]. In 2012, researchers used expressed sequence tag-simple sequence repeats (EST-SSRs) for genotyping to conduct a comparative analysis of orthologous groups between *Lilium*, and *Tulipa*, which revealed conserved genetic relationships and facilitated the identification of common EST–SSR markers [40]. Subsequently, EST–SSR markers were used to assess the genetic diversity of 280 individuals from 36 wild and cultivated samples collected from Iran and the Netherlands [149]. Similar studies using EST–SSR markers in the species *T. alberti* and *T. greigii* revealed high genetic variability within populations (75–77%) [60]. Overall, EST–SSR markers are highly informative and reproducible, gene-associated co-dominant markers that enable cross-species transferability and effective assessment of genetic diversity in *Tulipa* species. However, they generally exhibit lower polymorphism than genomic SSRs, provide limited genome coverage restricted to expressed regions, and therefore reduced resolution for genome-wide variability analyses [40,60,149].

Subsequently, based on a comparative analysis of plastid genomes, seven highly informative single-nucleotide SSR markers were proposed for use in population and phylogenetic studies [6]. In similar studies, population-level SSR marker analyses were also conducted for 15 populations of *T. buhseana* collected in two regions of Kazakhstan (Almaty and Zhambyl). Analysis of eight polymorphic markers revealed 31 alleles and showed a high level of genetic diversity within populations (66%) alongside significant inter-population differentiation (34%) [150]. For the first time, complete chloroplast genomes of four rare species (*T. alberti*, *T. kaufmanniana*, *T. greigii*, and *T. dubia*) were obtained and analyzed using SSR markers. Phylogenetic and molecular analyses identified 10 genes with highly polymorphic SSR markers for the identification, systematics, and further genomic studies of the genus *Tulipa* [2,151]. A comparative analysis of seven species (*T. behmiana*, *T. brachystemon* Regel (syn. *T. tetraphylla*), *T. kolpakowskiana*, *T. lemmersii*, *T. ostrowskiana*, *T. tetraphylla*, and *T. zenaidae*) growing in Kazakhstan revealed that 1388 SSR loci were detected in the plastids [41]. The results of this study may be used in future work to investigate phylogenetic relationships in depth and refine the molecular-taxonomic structure of species within the genus *Tulipa*. SSR markers revealed substantial intraspecific genetic diversity and significant population differentiation, while plastome-wide surveys identified thousands of SSR loci and several highly polymorphic genes useful for species identification and systematics. However, compared with SNP markers, plastid SSRs generally provide lower genomic resolution and reduced power to detect fine-scale genetic variation. Overall, plastid SSR markers represent a useful tool for resolving phylogenetic relationships and refining the molecular taxonomy of the genus *Tulipa*, particularly at broader evolutionary scales [2,6,41,150,151].

Single-nucleotide polymorphism (SNP) markers are widely used in research on cultivated *Tulipa* varieties. In 2012, work was carried out to genotype *Lilium* and *Tulipa*, revealing that conservative genetic links facilitated the identification of common SNP markers used for high-throughput genotyping in *Lilium* and *Tulipa* [40]. Furthermore, analysis of 236 SNP loci identified three genetic clusters among 72 samples from various cultivar groups, and 121 SNP markers were selected as effective for assessing genetic diversity, determining genetic relationships, and distinguishing *Tulipa* hybrids [152]. SNP markers are highly informative and are used in *Tulipa* research, enabling high-throughput analysis at genome-wide resolution to reveal genetic structure and diversity, as well as to determine relationships between species. However, their development requires prior genomic information and can be more expensive and resource-intensive, although they generally provide higher resolution for detailed genetic analysis [40,152].

In summary, no single marker system is sufficient to fully resolve genetic diversity and evolutionary relationships in *Tulipa*. Cytogenetic data provide a stable chromosomal framework but limited taxonomic resolution, while biochemical markers (e.g., esterase profiles) and RAPD show low reproducibility and weak discriminatory power. Dominant PCR-based markers (ISSR, AFLP, SRAP, iPBS, CDDP) are effective for detecting genetic polymorphism and population structure, but their inability to distinguish heterozygotes and variable resolution limit fine-scale analyses. Sequence-based markers (ITS, cpDNA, EST-SSRs) offer higher transferability and phylogenetic utility, yet are constrained by low variability, uniparental inheritance, or restricted genome coverage. Plastid SSRs improve resolution for phylogeography and taxonomy but remain less informative than SNP-based approaches, which provide the highest resolution for fine-scale population and evolutionary studies [40,152]. These data are of great importance for taxonomy and evolutionary biology, as well as for the development of strategies for the conservation of genetic resources, particularly endemic and IUCN Red List species, and for breeding of ornamental crops within the genus *Tulipa*.

## 5. Molecular Phylogenetics and Plastome-Based Genomics

The development of next-generation sequencing (NGS) and the availability of complete plastid genome sequences are increasingly important for reconstructing the evolutionary relationships of plants. Plastid data provide high-resolution phylogenetic information, particularly in complex taxonomic groups such as the genus *Tulipa* [6]. In parallel, the DNA barcoding approach is being actively developed, using conserved regions of the nuclear, plastid, and mitochondrial genomes, which is particularly important for identifying species that are morphologically difficult to distinguish. Among the most informative markers, the nuclear ITS region is widely used, as are plastid loci possessing high diagnostic value [74].

**Figure 2:**
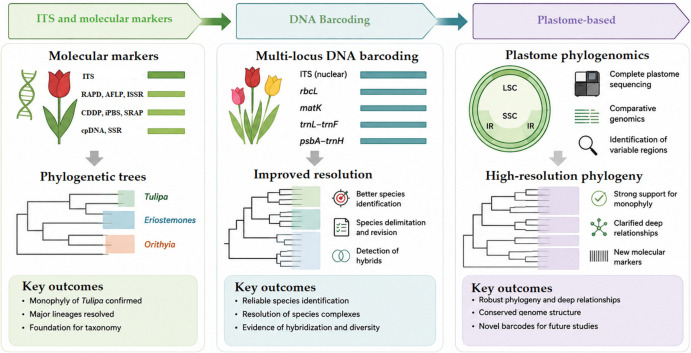
Overview of molecular markers and plastome-based approaches used in phylogenetic and taxonomic studies of genus *Tulipa*. The diagram illustrates the progression from traditional molecular marker analyses (**left**) through multi-locus DNA barcoding (**center**) to plastome-based phylogenomics (**right**). Green, blue, and purple colors denote successive methodological stages and do not represent specific taxa or phylogenetic relationships. Arrows indicate the chronological and methodological transition toward increasing genomic information content and phylogenetic resolution. Traditional molecular markers, including ITS, RAPD, AFLP, ISSR, CDDP, iPBS, SRAP, cpDNA, and SSR markers, have been used to infer phylogenetic relationships and major evolutionary lineages within genus *Tulipa*. Multi-locus DNA barcoding combines nuclear and plastid loci (ITS, rbcL, matK, trnL-trnF, and psbA-trnH) to improve species identification, species delimitation, and hybrid detection. Plastome phylogenomics utilizes complete chloroplast genome sequencing and comparative genomic analyses to resolve deep evolutionary relationships, identify informative molecular markers, and generate robust phylogenetic hypotheses.

Studies based on the analysis of nuclear and plastid markers have confirmed the monophyly of the genus *Tulipa* and revealed a stable phylogenetic structure comprising several major evolutionary lineages [19]. Further studies conducted on material from Turkey, using *trnL–trnF* and *ITS* markers, confirmed the division of the genus into three main clades (*Tulipa*, *Eriostemones*, and *Orithyia*). These markers demonstrated consistent phylogenetic signals, underscoring the importance of ITS for species-level analysis [153]. Phylogenetic analysis of the ITS region of nuclear ribosomal DNA clarified the systematic position of representatives of the genus *Tulipa* and proposed a new taxonomic structure comprising four subgenera and two sections. The study identified two main clades: the first united representatives of the subgenus *Orithyia*, while the second included the subgenera *Tulipa*, and *Eriostemones + Clusianae*. The subgenera *Eriostemones* and *Clusianae* formed two separate clusters. Subgenus *Eriostemones* is separated into two sections, *Biflores* and *Sylvestres* [154]. Separate studies also assessed the effectiveness of various DNA barcoding markers for species identification (Figure 2). Analysis of the *matK*, *psbA–trnH*, and *rbcL* markers showed that *matK* provides the highest resolution for identifying *T. edulis* [155]. Analysis of the chloroplast marker psbA-trnH revealed the genetic uniqueness of Ukrainian populations of *T. quercetorum* compared to *T. sylvestris*; however, further studies using nuclear molecular markers are needed to definitively determine their taxonomic status [156]. The use of the nuclear marker ITS and the plastid regions *trnL–trnF*, *rbcL*, and *psbA–trnH* revealed the division of eight *Tulipa* species into two main clades (*Eriostemones* and *Tulipa*). The study’s results also allowed revision of the species boundaries within the *T. scardica* complex and confirmed the synonymization of *T. luanica* and *T. kosovarica* with *T. serbica*. At the same time, the taxonomic status of *T. albanica* and *T. scardica* remains uncertain and requires further research [115]. As part of a study of the flora of Uzbekistan, a DNA barcoding approach was applied to the taxonomically complex *Tulipa* group, using plastid markers *(rbcL*, *psbA-trnH*, *matK*, *trnL-F*) and the nuclear ITS marker in 15 species (*T. greigii* Regel, *T. vvedenskyi*, *T. kaufmanniana*, *T. dubia*, *T. affinis*, *T. ferganica* Vved., *T. korolkowii*, *T. borszczowii*, *T. lehmanniana*, *T. intermedia*, *T. turkestanica*, *T. biflora*, *T. bifloriformis* Vved., *T. dasystemon*, *T. sogdiana*) and confirmed the effectiveness of genetic methods for the inventory and identification of species of the genus *Tulipa* [157]. Similar studies using three molecular markers (*psbA-trnH*, *trnL-trnF*, and ITS) were conducted on 15 wild Mediterranean, Balkan, or Greek native *Tulipa* species. This resulted in the construction of phylogenetic trees, that identified three main clades. The first group comprised representatives of the subgenus *Tulipa* (*T. scardica*, *T. undulatifolia*, *T. rhodopea*, *T. agenensis* and *T. raddii*), the second consisted solely of *T. clusiana* (subgenus *Clusianae*), and the third brought together representatives of the subgenus *Eriostemones*, including *T. bakeri*, *T. saxatilis*, *T. australis*, *T. cretica*, *T. goulimyi*, *T. orphanidea*, *T. bithynica*, *T. hageri* and *T. doerfleri* [134]. Analysis of ITS and plastid markers (*matK*, *ycf1b*) in *Tulipa* species from Aksu-Zhabagly revealed a division into two main clades. They confirmed the higher resolving power of ITS compared to plastid loci. However, *matK* proved highly conserved, whilst ycf1b evolved relatively slowly, limiting its variability while preserving its diagnostic value [74]. The combination of ITS and *rbcL*, *matK*, and *psbA–trnH* markers enabled the identification of new hybrid forms and further expanded our understanding of genetic diversity and evolutionary processes within the genus *Tulipa* [25]. Overall, the molecular genetic studies confirm the high effectiveness of ITS and plastid markers in reconstructing phylogenetic relationships and refining the systematics of the genus *Tulipa*. The data obtained indicate its monophyletic origin, reveal the main evolutionary lineages, and highlight the important role of hybridization in the group’s evolution. A comprehensive approach using DNA barcoding significantly improves species identification accuracy and provides a reliable basis for subsequent taxonomic and evolutionary studies.

In recent years, plastid genomes have been widely used to analyze taxonomically complex groups, including the genus *Tulipa*, which is characterized by high morphological variability. Comparative analysis of plastomes from different species, not only for the refinement of phylogenetic relationships but also for the identification of features of genomic structural organization levels, their level of conservatism, and promising molecular markers for DNA barcoding and systematics.

The complete chloroplast genome sequences of *T. altaica* were obtained from Zhou et al. [1]. The complete chloroplast genome is 146,887 bp in length and comprises a large single-copy region (LSC) of 78,433 bp, a small single-copy region (SSC) of 16,628 bp, and a pair of inverted repeats (IRa and IRb) of 25,913 bp each. The genome contains 111 unique genes, including 77 protein-coding genes, 30 tRNA genes, and 4 rRNA genes [1]. Furthermore, the genome of *T. iliensis* has been sequenced; it is 151,744 bp in length and contains 133 functional genes, whilst a phylogenetic analysis of sixteen species revealed its close relationship with *T. altaica* [42]. The circular chloroplast genome of *T. buhseana* is 152,062 bp in length and contains 133 functional genes. This species is most closely related to *T. altaica* [158]. Similar studies have been conducted for other species. The chloroplast genome of *T. patens* is 152,050 bp in length and contains 133 annotated functional genes, including 87 protein-coding, 38 tRNA, and 8 rRNA genes. Phylogenetic analysis of ten species revealed a close relationship between *T. patens* and *T. sylvestris* [18]. The chloroplast genome of *T. gesneriana* is 151,958 bp long and contains 126 genes. Phylogenetic analysis based on nine plastid genomes revealed a close relationship between this species and *T. iliensis* and *T. thianschanica* var. *sailimuensis* X.Wei & D.Y.Tan (syn. *T. iliensis*) [43]. Recent studies of the chloroplast genome of *T. sinkiangensis* have shown that it is 151,929 bp in length and comprises 136 genes, of which 87 encode proteins, 38 encode tRNAs, 8 encode rRNAs, and 3 are pseudogenes [44]. The sizes of the plastid genomes of *T. alberti* and *T. greigii* are 152,359 bp and 152,242 bp, respectively. A total of 136 genes have been annotated in their plastids, of which 114 are unique. These genes include 80 protein-coding genes, 30 tRNA genes, and 4 rRNA genes. The ycf1 gene region is highly variable and can be considered a promising molecular marker for DNA barcoding [45]. Phylogenomic analysis of plastid genomes of *T. altaica* (151,691 bp), *T. iliensis* (152,073 bp), *T. patens* (152,088 bp), *T. thianschanica* (152,087 bp), and *T. sylvestris* (151,940 bp) showed comparative plastome structure and evolutionary relationships among species. Phylogenetic analysis based on 24 plastid genomes strongly confirmed the monophyly of *Tulipa* and the sister relationship between *Tulipa* and *Amana* L. (*Erythronium*), *T. iliensis*, *T. thianschanica*, and *T. altaica* were grouped with *T. sylvestris* [6]. The phylogenetic analysis confirms robust phylogenetic relationships among the studied species, highlighting the informative value of plastid genomes for taxonomic research.

Thus, modern molecular genetic studies demonstrate the high informativeness of both nuclear and plastid markers for investigating the phylogeny, systematics, and genetic diversity of species within the genus *Tulipa*. Combining DNA barcoding data with whole chloroplast genome analysis enables more accurate reconstruction of evolutionary relationships and the refinement of the taxonomic status of individual species.

## 6. Challenges and Future Perspectives

One of the key challenges to conserving *Tulipa* diversity is climate change, which is considered a long-term threat to the IUCN Red List, rare and endangered species, and to primary and secondary centers of endemism [24]. Overgrazing poses an additional threat, leading to habitat degradation and a decline in the numbers of rare species [28]. Combined with other anthropogenic impacts, including land development and uncontrolled plant collection, this significantly increases the risk of degradation and extinction of natural *Tulipa* populations [5,9,119,159]. Predictive models indicate a significant reduction in suitable habitats and a decline in the effectiveness of the existing network of protected areas, highlighting the need for a transition to regionally focused and global conservation planning, as well as the development of adaptive biodiversity conservation strategies. Assessing the conservation status of species remains a serious problem, as relying solely on national criteria for assessing extinction risk does not always reflect a species’ actual status across its entire range, whereas global assessments are more informative and reliable [9,122,160]. At the same time, peripheral and remote populations should be considered when developing conservation programs, as they can play a vital role in preserving species’ genetic diversity and adaptive potential. An additional challenge is the transboundary distribution of many *Tulipa* species, whose ranges span multiple countries, highlighting the need for international cooperation and coordination of conservation measures to minimize the negative impacts of climate change and other anthropogenic factors [9,159].

A serious limitation is the lack of comprehensive data on many *Tulipa* species, including information on their distribution, ecology, and population sizes, which complicates an objective assessment of their conservation status [3,10]. Furthermore, taxonomic uncertainty, limited molecular data, and the limited research on some *Tulipa* species remain key challenges in current research [19,161]. This highlights the need to integrate genomic, morphological, ecological, and biogeographic approaches to better understand the evolution and effectively conserve of *Tulipa* biodiversity [3,10,19,161]. In this regard, a promising approach is to create a unified global database that combines taxonomic, genomic, ecological, and biogeographic information on all *Tulipa* species. A unique system will facilitate international collaboration, clarify species’ taxonomic status, and improve the effectiveness of conservation measures.

The next main challenge in *Tulipa* research remains the limited resolution of traditional phylogenetic approaches, which rely solely on a small number of plastid markers and the ITS region of the nuclear genome. Using a limited set of genetic data often complicates the reconstruction of evolutionary relationships and the delimitation of closely related species [25,162]. An additional challenge is that, despite a pronounced phylogenetic signal, genome size is insufficient to precisely differentiate taxa at the species level. Therefore, a promising direction is the use of integrative approaches combining data from whole plastome sequencing, DNA barcoding, nuclear and plastid markers, and morphological and ecological studies [6,41,115,134]. Analysis of large taxa samples and the use of whole-genome data significantly improve the accuracy of phylogenetic relationship reconstruction, clarify the taxonomic status of individual species, and more fully assess the genetic diversity of the genus *Tulipa*.

## 7. Conclusions

The genus *Tulipa* is a taxonomically complex group of plants, characterized by high morphological and genetic variability and by repeated revisions of its systematics. The primary center of *Tulipa* diversity is concentrated in the mountainous regions of Central Asia, whilst secondary centers of species formation are located in South-West Asia, the Eastern Mediterranean, and the Balkans. A comparison of various sources, including the Plants of the World Online database, reveals existing discrepancies in assessments of the genus’s species composition, reflecting the ongoing instability of its taxonomy. Recent studies demonstrate that integrating morphological, cytogenetic, and molecular-genetic approaches is the most effective way to study members of the genus. The use of various molecular markers, DNA barcoding methods, and whole chloroplast genome analysis significantly improves the accuracy of phylogenetic reconstruction and clarifies the taxonomic status of species. At the same time, the conservation of *Tulipa* biodiversity is complicated by the impact of human activities and climate change, which lead to habitat loss and the decline of wild populations. The lack of research on many recently described species, along with limited data on their distribution and ecology, underscores the need for further comprehensive studies. In this regard, integrating genomic, morphological, and ecological data is a key approach to refining taxonomy and developing effective strategies for the conservation of *Tulipa* genetic resources.

## Figures and Tables

**Table 3:** Comparative performance of marker systems used in *Tulipa* studies.

Marker System	Data Level	Main Application	Strengths	Limitations
Cytogenetic	Chromosomal	Taxonomy, karyotype analysis	Stable chromosome number; ploidy information	Low resolution
Biochemical (esterase)	Protein	Species differentiation	Simple, low cost	Low reproducibility
RAPD	DNA dominant	Genetic diversity	Fast, inexpensive	Poor reproducibility
ISSR	DNA dominant	Population structure	High polymorphism	Dominant, scoring bias
ITS	Nuclear DNA	Phylogeny	Biparental inheritance	Low variation in closely related taxa
cpDNA	Plastid DNA	Phylogeography	Conserved structure	Maternal inheritance
AFLP	DNA dominant	Intraspecific variation	High resolution	Complex, dominant
CDDP	Gene-targeted	Genetic relationships	Functional relevance	Low species discrimination
iPBS	DNA dominant	Population genetics	High polymorphism	Limited fine-scale resolution
SRAP	DNA dominant	Germplasm diversity	Informative clustering	Limited resolution
EST-SSR	Transcriptome SSR	Functional diversity	Co-dominant, transferable	Limited genome coverage
SSR	cp genome SSR	Taxonomy	Many loci	Lower resolution than SNPs
SNPs	Genome-wide	High-resolution genomics	Highest resolution	Cost, need for genomic data

## Data Availability

No new data were created or analyzed in this study. Data sharing is not applicable to this article.
